# Supplementary material for: GLP1 Exerts Paracrine Activity in the Intestinal Lumen of Human Colon
Source: Int J Mol Sci. 2022 Mar 24;23(7):3523. doi: 10.3390/ijms23073523 (PMC8998470; doi:10.3390/ijms23073523)
Supplement: Supplementary file 1 [file ijms-23-03523-s001.zip › ijms-1638405-supplementary.pdf]

International Journal of
Molecular Sciences

MDPI

Supplementary materials

Cell culture:

Cells were maintained in 75 cm^2 culture flasks (Greiner Bio-One, Frickenhausen, Germany) at 37°C in an atmosphere of 5% CO_2 with the medium being changed every 2–3 days. The growth medium for Caco-2 cells consisted of DMEM supplemented with 10% (v v 1) heat-inactivated FBS, 2 mmol L^{-1} L glutamine, 1 mol L^{-1} HEPES and 100 U mL^{-1} penicillin-streptomycin mixture. When confluence reached ~80%, Caco-2 cells were harvested by treatment with 0.25% Trypsin– 1 mol L 1 EDTA for 10 min, and then split and sub-cultured in fresh growth medium. The growth medium for Raji-B and THP-1 cells consisted of RPMI 1640 supplemented as described above. Both Raji-B and THP-1 cells were maintained at a density between $1×10^5$ and $1×10^6$ cell mL^{-1}. When density reached $1×10^6$ cell mL-1, cells were split 1:10 and then sub-cultured in fresh growth medium.

We used three different culture-based models of gut epithelium: a Caco-2 cell monoculture, a Caco-2/THP-1 cell co-culture and a Caco-2/Raji-B/THP-1 tri-culture. The co- and tri-culture models were set up by adapting the protocol described by des Rieux et al. (45) for co-cultured Caco-2/Raji-B cells as follows: 0.5-mL aliquots of Caco-2 cell suspension ($5×10^5$ cells mL^{-1}) were added in permeable transwell inserts (1.1-cm^2 surface area and 3-µm pore size) in 12-well plates (Merck Millipore, Darmstadt, Germany). One mL of DMEM growth medium was added to the wells and both apical and basolateral media were changed every other day. On day 5, inserts were inverted over a culture dish (245 mm×245 mm×20 mm) filled with DMEM growth medium. Pieces of silicon tubes were then placed on the basolateral sides and filled with DMEM growth medium. Inverted inserts were cultivated for 11 days and the basolateral media were changed every other day. On day 16, 0.4-mL aliquots of Raji-B cell suspension ($5×10^5$ cells mL^{-1} in DMEM:RPMI (2:1) growth medium) were added to the basolateral compartments and cultured for five additional days. On day 21, the silicon tubes were removed, and the inserts were washed twice with HBSS. Inserts where then placed in 12-well plates with fully differentiated THP-1 in each well. THP-1 monocytes ($5.5×10^5$ cells per well) were differentiated into adherent macrophage-like cells by treatment with PMA (100 ng mL-1 in RPMI growth medium without FBS) 48 h prior to the experiments.

Western Blot procedure

Proteins from human colon mucosa, adipose tissue and heart were extracted using RIPA buffer (20mM Tris, pH 7.4, 150mM NaCl, 0.5% Triton-100, 1mM Na3VO4, 1mM PMSF). Then, SDS-PAGE electrophoresis with stain-free gels (Bio-Rad) was performed to separate thirty micrograms of proteins per sample, which were then transferred to PVDF membranes with Trans-Blot® TurboTM (Bio-Rad). Membranes were incubated overnight at 4°C with primary antibodies (Table 1). Next, membranes were incubated for 2 hours at room temperature with peroxidase-conjugated secondary IgGs. Images were acquired and analysed with Image Lab software on a ChemiDoc TM Touch instrument (Bio-Rad), using fluorescence emission of protein bands separated on stain-free gels for total lane normalization.

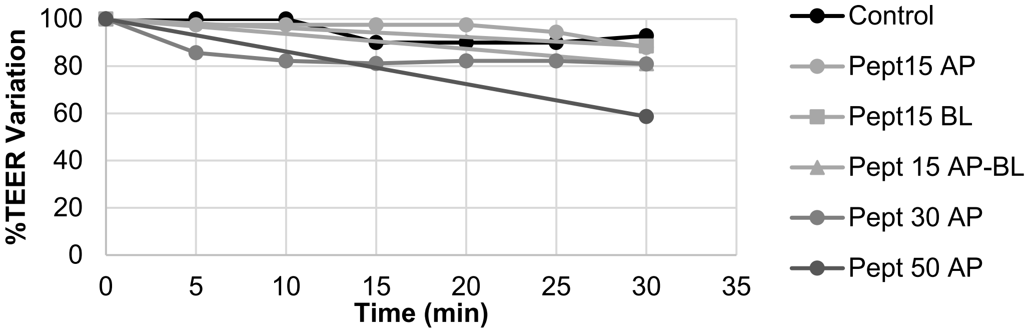

Scheme S1. Effect of 30 minutes meat apical peptone treatment in human descending colon. Effect of meat peptone at different concentrations (15, 30 or 50 mg/ml) on TEER (C) (n=4-7).

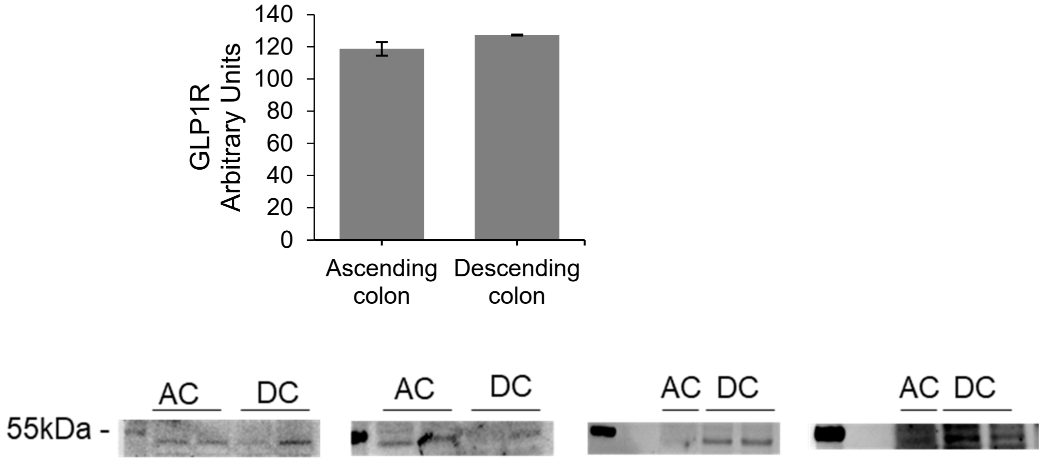

Scheme S2. GLP1R expression in human ascending and descending colon detected by Western Blot Quantity of GLP1R in each lane was normalized on stain-fee gel total protein content quantification. AC: Ascending colon; DC: Descending colon. Data are expressed as mean ± SEM GLP1R expression in n=4 tissue samples.

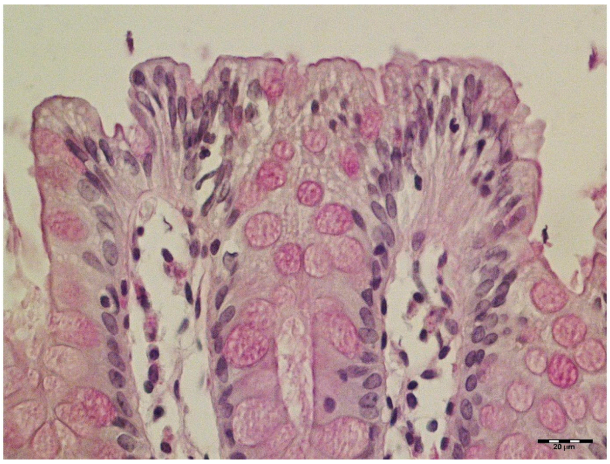

Scheme S3. PAS staning of mucus (pink) in goblet cells of human colon mucosa.

A B

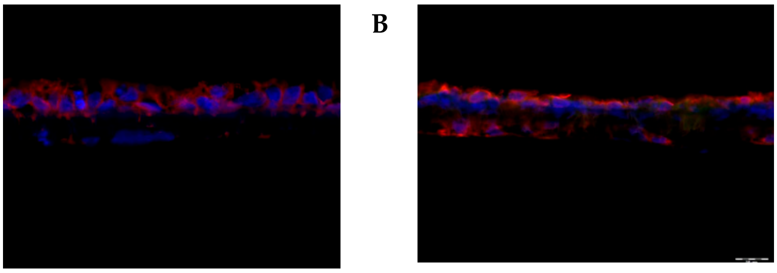

Scheme S4. GCGR and GLP1 expression in Caco2+THP1 cell co-culture. (A) GCGR (red) expression. (B) GCGR (green) and GLP1 (red) expression in Caco2+THP1 cells. Representative of n=4 preparations from independent models. Nuclei were stained with DAPI (blue).

Table S1. Antibodies used for Western Blot (WB) and Immunofluorescence (IF) analyses.

Antibody	Ref.	Dilution
1ary Anti-GLP1R (mouse)	sc-390773, Santa Cruz Biotechnology	1:1000 WB<br>1:100 IF
1ary Anti-Actin (goat)	sc-1615, Santa Cruz Biotechnology	1:100 IF
1ary Anti-GCGR (rabbit)	ab75240, Abcam	1:250 IF
2ary peroxidase-conjugated IgG	Southern Biotech #2010-05	1:2000 WB
2ary Alexa green 488 (anti-rabbit)	AffiniPure donkey, Jackson ImmunoResearch Europe	1:175 IF
2ary Alexa green 488 (anti-goat)	AffiniPure sheep, Jackson ImmunoResearch Europe	1:175 IF
2ary Alexa red 594 (anti-mouse)	AffiniPure sheep, Jackson ImmunoResearch Europe	1:175 IF
2ary Alexa red 594 (anti-rabbit)	AffiniPure sheep, Jackson ImmunoResearch Europe	1:175 IF

Table S2. Patient characteristics.

Clinical characteristics	Number of patients	Percentage (%)
Gender		
Male	9	47.4
Female	10	52.6
Colon segment		
Ascending	11	57.9
Descending	8	42.1
Hypertension	10	52.6
under treatment	9	90.0
Dyslipidemia	6	31.6
under treatment	5	83.3
Diabetes Mellitus type II	5	26.3
under treatment	5	100.0
**Clinical characteristics**	**Mean ± SEM**	
Age (years)	66.3 ± 1.8	
BMI	26.9 ± 1	
Blood glucose (mM)	5.8 ± 0.4	
Blood cholesterol (mM)	3.1 ± 0.3	
